# Effect of agricultural diversification on dietary diversity in rural households with children under 5 years of age in Zambia

**DOI:** 10.1002/fsn3.2587

**Published:** 2021-09-17

**Authors:** Chewe Nkonde, Keiron Audain, Rebecca N. Kiwanuka‐Lubinda, Pamela Marinda

**Affiliations:** ^1^ Department of Agricultural Economics & Extension School of Agricultural Sciences University of Zambia Lusaka Zambia; ^2^ Department of Food Science & Nutrition School of Agricultural Sciences University of Zambia Lusaka Zambia

**Keywords:** agricultural diversification, children, dietary diversity, policy, rural households, stunting

## Abstract

Micronutrient deficiencies in low‐income countries are associated with the monotonous consumption of nutrient‐deficient crops, contributing to childhood stunting with far‐reaching socioeconomic consequences. To promote nutrition sensitive agriculture, policy makers in such countries have embarked on policy initiatives that encourage agricultural diversification in smallholder farming systems. This paper investigates the link between agricultural diversification and two key indicators of food and nutrition security among children under 5 years in rural Zambia. Data from the 2015 Rural Agricultural Livelihoods Survey and regression models are used to explain household dietary diversity and months of inadequate household food provisioning among 7934 households. Factors associated with the key outcome variables include land cultivated, household size, total livestock units, household head education, households receiving extension information, and use of productivity‐enhancing inputs such as fertilizers. Although the results demonstrate that agricultural diversification is positively associated with the household dietary diversity score, the relationship is not statistically significant. Further, the study findings illustrate that agricultural diversity is negatively associated with months of inadequate household food provisioning but that this relationship is also not statistically significant. The implication for policy is that other interventions such as productivity enhancement and behavioral change communication need to be scaled up.

## Graphical abstract

Micronutrient deficiencies in low‐income countries are associated with monotonous consumption of nutrient‐deficient crops, which contributes to childhood stunting. This paper investigates the link between agricultural diversification and dietary diversity among households with children under the age of 5 years in rural Zambia.

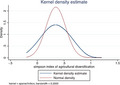

## 1 INTRODUCTION

Approximately 2 billion people globally are affected by micronutrient deficiencies; much of which is attributed to consuming a monotonous diet of nutrient‐deficient staple crops (Haas et al., 2016; Ritchie & Roser, 2017). While monocrop cultivation has led to increased food production and calorie consumption, the over‐reliance on a few major crops is linked to monotonous diets and micronutrient deficiencies (Jones et al., 2017). This, coupled with the effects of climate change, has perpetuated poor diet quality and the proliferation of diet‐related diseases (Tan et al., 2020). Ironically, most people experiencing micronutrient deficiencies belong to smallholder farming communities in rural areas (Sibhatu et al., 2015). In such communities, wasting in children under 5 years can be up to nine times higher than the national prevalence. Micronutrient deficiencies are also noted in young children and can lead to stunting, which can be up to four times higher in vulnerable communities (Mulmi et al., 2017; UNICEF, 2020).

According to the 2018 Zambian Demographic Health Survey (ZDHS), approximately 35% of Zambian children are reportedly stunted (Central Statistical Office [CSO], 2019). In 2019, the Global Hunger Index (GHI) ranked Zambia 113th out of 117 qualifying countries, with a score of 38.1, signaling alarming levels of hunger (GHI, 2019). The GHI ranks countries based on undernourishment, child mortality, child wasting (low weight for height), and child stunting (low height for age). In Zambia, the underfive mortality rate between 2014 and 2018 was estimated at 61 deaths per 1000 births (CSO, 2019). Acute and chronic micronutrient deficiencies are highly prevalent, particularly in rural areas. For instance, the prevalence of some degree of anemia among children under 5 years was approximately 58% based on hemoglobin levels in grams/decilitre, with 29% of children classified as mildly anemic (10.0–10.9 g/dl), 28% were moderately anemic (7.0–9.9 g/dl), and 2% were severely anemic (<7.0 g/dl) (ZDHS, 2019). The consequence of micronutrient deficiencies is poor cognitive development, which has far‐reaching social and economic consequences (Mulmi et al., 2017). To avoid this, children should be fed a diverse diet consisting of various nutrient‐rich foods (Mulmi et al., 2017). It has been proposed that promoting agricultural diversification among farming households can improve dietary diversity and hence the nutritional status of children (Mofya‐Mukuka & Kuhlgatz, 2016).

In Zambia, the government has embarked on policy initiatives to promote agricultural diversification and reduce the dependence on maize, Zambia's most commonly grown food crop. This involves cultivating various crops such as cassava, sweet potato, groundnut, sunflower, and soya beans (Sichoongwe et al., 2014). Diverse crop cultivation can boost productivity and improve the stability of agroecosystems, whereas a lack of diversification can have a negative knock‐on effect on global diet quality (Jones, 2017). Agricultural diversification can help ensure food security by improving farmer adaptability and reducing vulnerability, which is crucial, given the predicted climate changes and the heavy reliance of smallholder farmers on rain‐fed crops. Farmers can thus avert risk and increase/improve income streams by adopting diversification practices.

Despite agricultural diversification being among the key policy strategies promoted to ensure nutrient adequacy and overall household food security, the link between agricultural diversification and household dietary diversity remains inconclusive and needs to be supported by evidence‐based research (Jones, 2017; Sibhatu et al., 2015).

Due to the paucity of data, it remains unclear whether agricultural diversification directly impacts dietary diversity within agricultural households, as it may be affected by other sociodemographic factors and household characteristics. While higher income levels, education, and nonfarm enterprise engagement may strongly stimulate adequate nutrient intakes, increases in the number of adolescents would substantially diminish it as the household would have more individuals to feed and given the additional nutritional requirements of adolescents (Akerele et al., 2017). The purpose of this paper is to estimate the effect of agricultural diversification on household dietary diversity across the distribution of rural households with children under the age of 5 years.

## 2 METHODOLOGY

### 2.1 Data

The study used secondary data from the 2015 Rural Agricultural Livelihoods Survey (RALS). In 2015, the RALS of small and medium‐scale farming households was conducted in Zambia by the Indaba Agricultural Policy Research Institute (IAPRI) in conjunction with the CSO of Zambia and the Ministry of Agriculture (MoA). The RALS is a longitudinal survey that utilized a new sampling frame derived from the 2010 census. The survey design followed a two‐stage stratified sampling frame where at the first stage, standard enumeration areas (SEA) were selected using the probability proportional to size sampling scheme. At the second stage, households were randomly sampled from the listed households in each SEA. A total of 7934 households were interviewed. The survey collected data on several questions related to the following main themes: demographic characteristics of household members; farmland and use; crop sales from own production; input and credit acquisition; livestock ownership and marketing, and off‐farm income sources. Concerning agriculture, households surveyed for the RALS provided detailed reporting on the amount and frequency of crops harvested for 2015, including the amount of livestock raised.

#### 2.1.1 Key treatment variable

Our treatment variable was the agricultural diversification index. *Agricultural diversification* is defined as a shift from a monocrop culture toward producing numerous crops, thus allowing farmers to have produce to sell and consume throughout the year. It also incorporates the promotion of fisheries and livestock for both income generation and regular consumption (Ministry of Agriculture and Ministry of Livestock and Fisheries, 2016). Our treatment variable was the agricultural diversification index. To measure the household level of agricultural diversification, we adapted the Simpson Index (SI) used by Basavaraj et al. (2016). The SI was computed as follows:

$$\text{SI}=1‐\sum P_{\mathrm{ij}}^{2}$$

where *P_ij_* = *A_ij_*/∑*A_j_* is the proportion of the ith agricultural enterprise value of output (crop or livestock) of total agricultural value of output by household *j*. If SI is near zero, it indicates that a household is highly specialized. If it is close to one, then the household is fully diversified in terms of agricultural production.

### 2.2 Key outcome variables

To explore the effect of agricultural diversification on dietary diversity and nutrient adequacy across households with children under 5 years, two outcome variables were considered in the current study. These were household dietary diversity score (HDDS) and months of inadequate household food provisioning (MIHFP). We describe these in turn.

Household dietary diversity score accounted for the number of different foods or food groups consumed over a 24 h period. In the RALS data set, dietary diversity was estimated using data collected during a 24 h recall period rather than a seven‐day time frame. Although longer recall periods capture a wider variety of foods consumed by a household, a 24‐h recall period is beneficial because it increases estimation accuracy by reducing the level of “noise” added. Dietary diversity and agricultural diversification (for rural households) were compared with the age and income activities of household head.

Household dietary diversity score is one of the most commonly used dietary diversity indicators at household level (Headey & Ecker, 2013). It accounts for the number of different foods or food groups consumed over a reference period, usually ranging from a day (24 h) up to 2 weeks (Heady & Ecker, 2013; Ruel, 2003). The HDDS was developed by the Food and Nutrition Technical Assistance (FANTA) Project of the United States Agency of International Development (USAID) with a maximum score of 12 food groups (Swindale & Bilinsky, 2006a; 2006b). The RALS 2015 was used to construct the HDDS outcome variable for this paper. To elicit information about household dietary diversity, the RALS questionnaire asked respondents about food products (organized into 16 categories) consumed by household members during the 24‐h recall period prior to the survey. Our study aggregated some of the food groups captured by the RALS data—those associated with vegetables, fruits, and meats—to construct a HDDS indicator consistent with the 12 food groups identified by the USAID‐FANTA project.

MIHFP was used to measure household food provisioning as a proxy measure of household food access. According to Deitchler et al. (2010), over time, the MIHFP indicator can capture changes in the household’s ability to address vulnerability in such a way as to ensure that food is available above a minimum level throughout the year. Measuring the MIHFP has the advantage of capturing the combined effects of a range of interventions and strategies, such as improved agricultural production, storage, and interventions that increase the household's purchasing power.

We used the RALS data to also generate this variable. During the survey, households were asked to indicate the months they did not have enough food to meet their needs during a 12‐month recall period. A household with inadequate food or food needs during a particular month was assigned a value of one, otherwise zero. The household‐level MIHFP indicator was calculated by summing all the values for the whole recall period. Households with no food needs had a zero value, and those with acute food needs had twelve.

### 2.3 Empirical strategy

The empirical goal for our paper was to measure the association between dietary diversity and agricultural diversity across households with children <5 years, ceteris paribus. The structural model takes the following form:

$$Y_{i}\;=\;\beta_{1}\;+\;\beta_{2}AgDiv_{i}\;+\;\beta_{3}Under5_{i}\;+\;\beta_{4}AgDiv\;*\;Under5_{i}\;+\;Z_{i}\beta_{5}\;+\;\varepsilon_{i}$$

where *Y_i_* represents a vector of the two measures of dietary diversity (HDDS and MIHFP) for each household *i; AgDiv_i_* is the SI for agricultural diversity at household level; *Under*5_i_ is the tercile categorical variable for the number of children under 5 years at household level; AgDiv*Under5_i_ is an interaction term between the agricultural diversity index and the categorical variable for number of children under 5 years for each household*; Z_i_* is a vector of exogenous variables such as household‐level characteristics and attributes related to agricultural production; the *β*'*s* are the parameter estimates; and ε*_i_* is the error term.

The empirical strategy for establishing the relationship between agricultural diversification and alternative measures of dietary diversity in this paper was econometrically specified in two ways. First, we specified the model using ordinary least squares regression where each measure of dietary diversity was regressed on explanatory variables based on the assumption that our key explanatory variable (*AgDiv*) is uncorrelated with the error term. Second, we estimated equation (2) by using an approach that accounts for potential sample selection bias likely to be imposed by violating the assumption that diversification is uncorrelated with the error term.

According to Mazunda et al. (2015), the link between agricultural diversification and dietary diversity is wrought with various unobservable characteristics. Important differences in food security and nutritional outcomes will likely exist between households that choose to diversify their crops and livestock production and those that do not. However, not all observed improvements in outcomes can be directly attributed to the decision to diversify agriculture since there could be other factors—the quality of land, risk, and food preferences—contributing to this and thereby improving food security and nutritional outcomes. This complexity in the linkage poses an endogeneity concern in that any observed relationship between agriculture diversification and the outcomes of interest may be due to any of these factors. To circumvent this potential pitfall, we used the Heckman two‐step procedure.

As the name implies, the procedure is in two stages. In the first stage (selection equation), probit analysis is used to identify what determines the decision by a household whether to diversify agricultural production or not. The selection equation is specified here below:

$$z_{i}^{*}=\gamma^{\prime }w_{i}+u_{i}$$

$$\text{where}\;u_{i}\;∼\;N\left(0,1\right)\;\text{and},$$

$$z_{i}\;=\;1\;\;\text{if}\;z_{i}^{*}\;>\;0,$$

$$z_{i}\;=\;0\;\text{if}\;z_{i}^{*}\;\leq \;0$$

The selection variable $z_{i}^{*}$ is not observed but rather a sign of whether or not household *i* diversified agricultural production (we explain how this variable was constructed in the next section), *w* is a vector of factors influencing the decision to diversify, *γ*’s are the parameter estimates, and *u* is the error term which is normally distributed with mean zero and unit variance. The second stage (outcome equation) measures the association between each indicator (HDDS and MIHFP) and agricultural diversity, while controlling for other confounding factors. The outcome equation is similar to the structural model (equation 2) except that we impose exclusion restrictions to allow for more robust identification.

To account for exclusion restrictions, we used the approach proposed by Di Falco et al. (2011). To proceed, we first estimated a probit model for the whole sample on variables conjectured to determine whether a household diversified agricultural production. Next, an ordinary least squares (OLS) regression was estimated for each outcome variable (HDDS and MIHFP). The right‐hand side variables only included the significant variables from the probit model estimated earlier. The variables not statistically significant in the second stage (OLS) constituted possible candidates for exclusion restrictions.

The RALS data set was checked for relevant variables of interest for analysis. Data utilized in the analysis included socioeconomic, demographic, and consumption data. Descriptive analysis of the data was done using SPSS Version 20, while econometric analysis used Stata 15.

## 3 RESULTS AND DISCUSSION

### 3.1 Descriptive statistics

Table 1 shows the descriptive statistics of the sampled households. A total of 7467 (94.2%) farmers resided in rural areas. The population can be predominantly characterized as farmers from rural households with at least one child under 5 years of age. Most household heads are educated at least up to the primary school level. The highest percentage of farmers were from the Eastern province (*n* = 2061; 26.0%), with the lowest from Lusaka (*n* = 446; 5.6%). The mean number of hours to the nearest urban center with at least 500,000 inhabitants was 13.40 (*SD* = 8.57).

**TABLE 1:** Descriptive analysis of household characteristics and sociodemographic factors

Variable	Mean	Standard deviation	Min	Max
Household dietary diversity score	5.87	2.14	0	12
Months of inadequate food provisioning	3.68	1.74	1	12
Agricultural diversification	0.34	0.18	0	1
Age of children under five	3.04	1.25	0	5
Landholding size (ha)	3.01	3.29	0	67.5
Education of household head (years)	8.04	3.44	0	19
Age of household head	48.64	14.87	16	105
Hectares cultivated (ha)	2.41	2.44	0	45.5
Number of children under five	1.56	0.74	1	8
Crop diversification	0.40	0.23	0	0.84
Distance to nearest urban center (h)	13.40	8.57	0.42	57.00
Total quantity of inorganic fertilizer used (kg)	302.28	562.00	0	10,400
Off farm income (Zambian Kwacha)	11,838.22	39,088.07	0	1,095,000
Total Livestock Units owned	3.65	10.3	0	250

Note

1 US Dollar = approx. 16 Zambian Kwacha (2021).

A total of 78.8% (*n* = 6251) of households were male‐headed, while 21.2% (1674) were female‐headed. The mean age of farmers was 48.64 years. Every household had at least one child under 5 years of age, with one household having eight children. The mean number of children per household was 1.56 (*SD* = 0.74). The mean age of children under five was 3.0 years (*SD* = 1.24). Regarding education of the household head, 12.7% (*n* = 1011) had no formal education; 20.1% (*n* = 1595) attended lower primary school (grades 1–4); 37% (*n* = 2932) attended upper primary school (grades 5–7); 26% (*n* = 2070) attended secondary school (grades 8–12); and 3.9% (309) attended postsecondary school. The highest proportion of mothers were educated up to Grade 7/Standard 6 16.1%; *n* = 1278), followed by no education (15.3%; *n* = 1215).

Regarding cultivated land, the mean size was 2.41 hectares (*SD* = 2.43). According to the SI for agricultural diversification (where 1 is most diversified, and 0 is not diversified), 46.5% (*n* = 3689) of farmers had diversification levels >0.6; 39.9 (*n* = 3165) had levels between 0.3 and 0.6; and 13.5% (*n* = 1071) had levels <0.3. The mean SI for agricultural diversification was 0.35 (*SD* = 0.18). The mean number of months households went with inadequate food provisions was 3.68 (*SD* = 1.74).

A total of 79.2% (*N* = 6277) of farmers owned their own livestock. The mean value of Tropical Livestock Unites was 3.65 (*SD* = 10.30). The mean total of fertilizer used was 302.28 kg (*SD* = 562.00). A total of 16.3% of farmers irrigated some fields. A total of 60.1% (*n* = 4765) of farmers received crop diversification related extension advice and 39.8 (*n* = 3160) did not, whereas 41.6% (3298) of farmers received FISP inputs and 58.3% (*n* = 4627) did not. Conservation tillage was adopted by 14.5% (*n* = 1148) of farmers, while 85.4% (*n* = 6777) did not. Regarding household dietary diversity, the mean HDDS was 5.87 (*SD* = 2.14).

Independent sample *t* test analysis on provincial data on HDDS, MIHFP, and agricultural diversification revealed that the Western province fared the least in all three variables (Table 2). Results showed a significant difference in HDDS between Copperbelt with the highest score of 6.86 and the Western Province with the lowest score of 4.53 (mean difference: 2.33).

**TABLE 2:** Provincial mean differences in HDDS, MIHFP, and agricultural diversification

Province	Household dietary diversity score (HDDS)	Months of inadequate household food provisioning (MIHFP)	Agricultural Diversification
Central	6.33 ± 2.11(*n* = 650)	3.25 ± 1.44 (*n* = 190)	0.35 ± 0.17(*n* = 650)
Copperbelt	6.86 ± 2.26 (*n* = 549)	3.27 ± 1.15 (*n* = 227)	0.32 ± 0.19 (*n* = 549)
Eastern	6.16 ± 1.99 (*n* = 2061)	3.08 ± 1.36 (*n* = 839)	0.40 ± 0.17 (*n* = 2061)
Luapula	5.75 ± 2.02 (*n* = 692)	4.01 ± 1.72 (*n* = 346)	0.41 ± 0.18 (*n* = 692)
Lusaka	6.56 ± 2.23 (*n* = 446)	3.28 ± 1.44 (*n* = 127)	0.31 ± 0.19 (*n* = 446)
Muchinga	5.48 ± 2.02 (*n* = 713)	3.95 ± 1.73 (*n* = 378)	0.34 ± 0.18 (*n* = 714)
Northern	5.05 ± 1.93 (*n* = 790)	3.81 ± 1.77 (*n* = 354)	0.37 ± 0.18 (*n* = 790)
North Western	5.83 ± 2.07 (*n* = 515)	3.69 ± 1.99 (*n* = 152)	0.34 ± 0.20 (*n* = 515)
Southern	6.01 ± 2.16 (*n* = 892)	4.05 ± 2.04(*n* = 344)	0.27 ± 0.15 (*n* = 892)
Western	4.53 ± 193 (*n* = 616)	4.72 ± 1.99 (*n* = 327)	0.23 ± 0.19 (*n* = 616)

Regarding months of inadequate food provisions, there was a significant difference in MIHFP between Eastern and Western province (1.64 + 0.11 (*p* < .01). Luapula province showed the highest agricultural diversification (0.41 + 0.18) and Western province the lowest (0.23 + 0.19); however, there was no significant difference between both provinces (0.19 + 0.01) (Table 3).

**TABLE 3:** Frequencies of household characteristics and sociodemographic factors (*n* = 7925)

Variable	Frequencies	Percentages
Sex of household head	Male = 6251 Female = 1674	Male = 78.8% Female = 21.2%
Received extension advice on diversification	Yes = 4765 No = 3160	Yes = 60.1% No = 39.8%
Practiced conservation tillage	Yes = 1148 No = 6777	Yes = 60.1% No = 39.8%
Education of mother	Standard 6/Grade 7 = 1278 None = 1215 Standard 4/Grade 5 = 699	Yes = 60.1% No = 39.8%
Education of household head	None = 1011 Primary (1–4) = 1595 Primary (5–7) = 2932 Secondary (8–12) = 2070 Postsecondary = 309	None = 12.7% Primary (Grade 1–4) = 20.1% Primary (Grade 5–7) = 37% Secondary (Grade 8–12) = 26% Postsecondary = 3.9%

In Table 4, “Households with diversified diets” were defined as households that consumed six or more food groups following the HDDS. “Educated mothers” were defined as mothers that received at least a primary school education. Households with more and less than six months of adequate food provisions were calculated accordingly. According to Pearson chi‐squared results, all the characteristics compared (male household heads, recipients of extension advice on diversification, practicing conservation tillage, and educated mothers) were significantly associated with households having a diversified diet and having more than six months of adequate food provisions. A double‐hurdle model analysis of secondary data from the Zambian Central Statistical Office revealed that the type of tillage technique used by farmers strongly influenced crop diversification (Sichoongwe et al., 2014).

**TABLE 4:** Comparison of means of selected characteristics between households with and without diversified diets and those with adequate and inadequate food provisioning

Household characteristic	Overall	HHs with diversified diets	HHs without diversified diets	Pearson chi^2^, χ^2^	Overall	HHs with more than 6 months food provisioning	HHs with less than 6 months food provisioning	Pearson chi^2^
Male household head	*n* = 6250	56.34% *n* = 3521	43.66% *n* = 2729	64.96* (*p* =.00)	*n* = 2428	93.37% *n* = 2267	6.63% *n* = 161	12.76* (*p* =.00)
Received extension advice on diversification	*n* = 4765	60.78% *n* = 2896	39.22% *n* = 1869	220.93* (*p* =.00)	*n* = 1808	94.69% *n* = 1712	5.31% *n* = 96	30.33* (*p* =.00)
Practiced conservation tillage	*n* = 1148	59.93% *n* = 688	40.07% *n* = 460	19.00* (*p* =.00)	*n* = 405	95.06% *n* = 385	4.94% *n* = 20	4.69* (*p* =.03)
Educated mother	*n* = 5757	55.81% *n* = 3213	44.19% *n* = 2544	116.03* (*p* =.00)	*n* = 2271	92.82% *n* = 2108	7.18% *n* = 163	9.92 (*p* =.002)

* Significant.

Agricultural production diversity appeared to be positively associated with household dietary diversity. It is therefore possible that when other crops are grown alongside maize, household food security may increase through access to a more diverse range of foods.

Although the gender of the household head appeared to have no association with agricultural diversity, male‐headed households had higher household dietary diversity scores (HDDS) and fewer months of inadequate food provisions compared to female‐headed households. Male farmers have traditionally had better access to resources that allowed for improving farm income, and better opportunities to earn off‐farm income, which improves access to more food choices by allowing the purchase of diverse foods for home consumption.

Results indicate that access to inputs and extension advice plays a significant role in achieving agricultural diversification. Receiving extension advice and the practice of conservation tillage may have also contributed to improving household dietary diversity and reducing the number of months households had inadequate food provisions.

Despite most farmers receiving extension advice services, only a tiny fraction was practicing conservation tillage. Both the mean HDDS and agricultural diversification score were below 50%, indicating inadequate household dietary diversity and levels of diversification.

#### 3.1.1 Distribution of the agricultural diversification inde*x*

**FIGURE 1:**
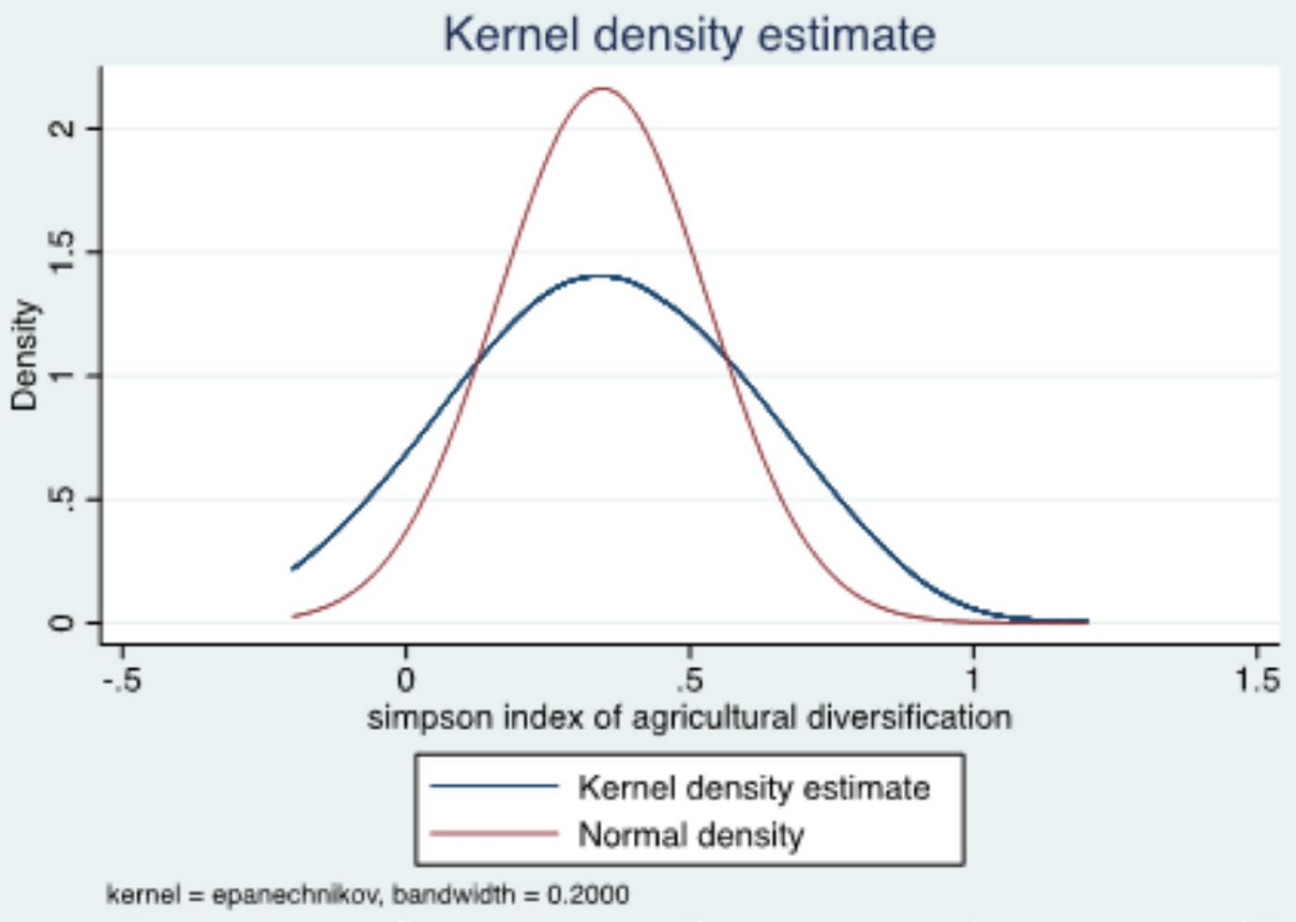
Distribution of the agricultural diversity index across households

Figure 1 presents the distribution of our key explanatory variable, agricultural diversity, across the sampled households using the kernel density estimate overlaid by a fitted normal density. Although the kernel density plot is less peaked than the normal density plot, the graph clearly shows that it is not skewed. Given this, the mean for the SI of agricultural diversification is used as the cutoff point to create a binary variable of whether a household diversified its agricultural activities. Households below the mean of the diversification index were assigned a value of zero (did not diversify), and those above the mean are assigned a value of one (completely diversified). This binary variable is used as a dependent variable in the selection equation for our econometric results discussed later.

#### 3.1.2 Bivariate relationships between dietary diversity and agricultural diversity

**FIGURE 2:**
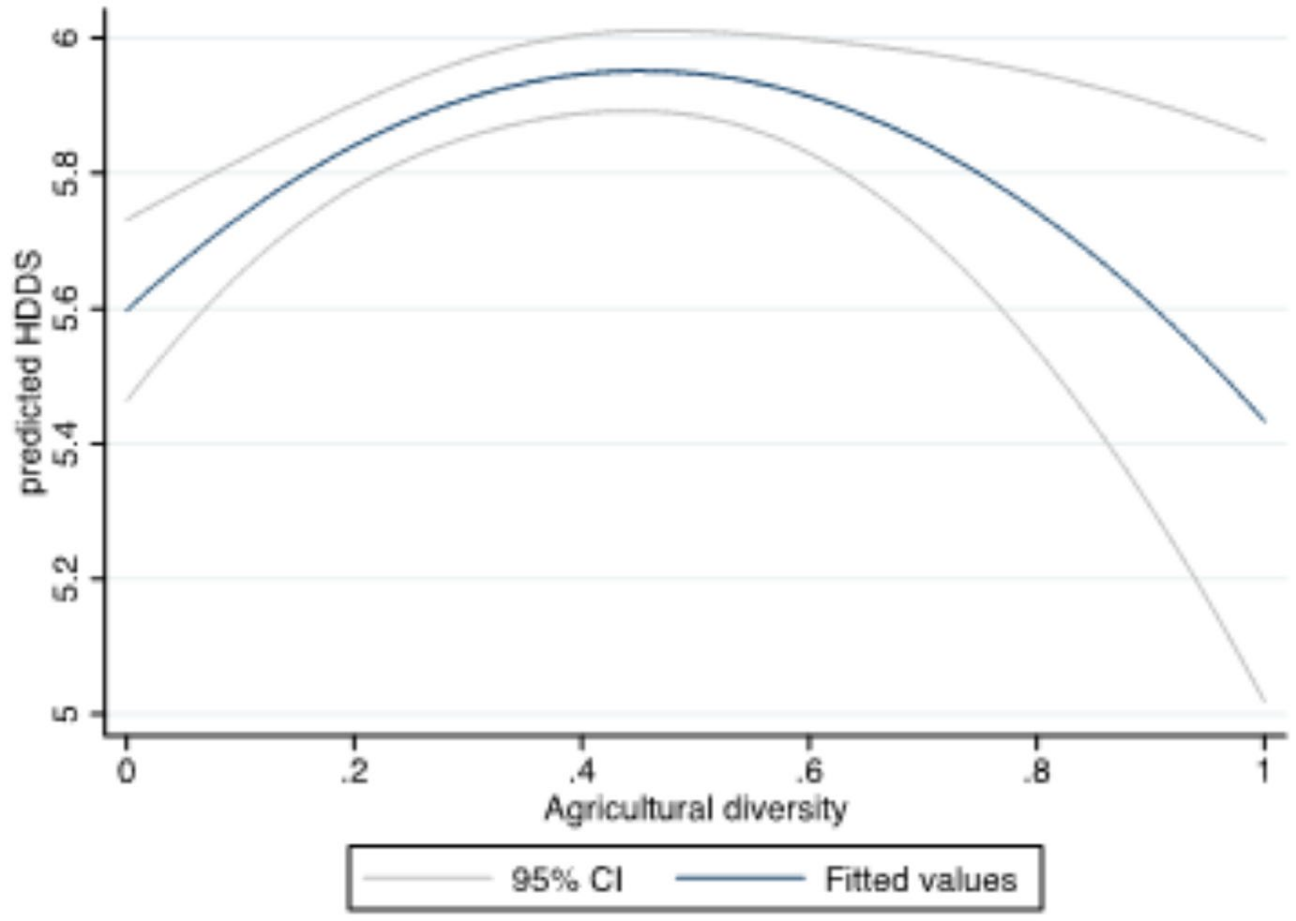
Relationship between HDDS and agricultural diversity

**FIGURE 3:**
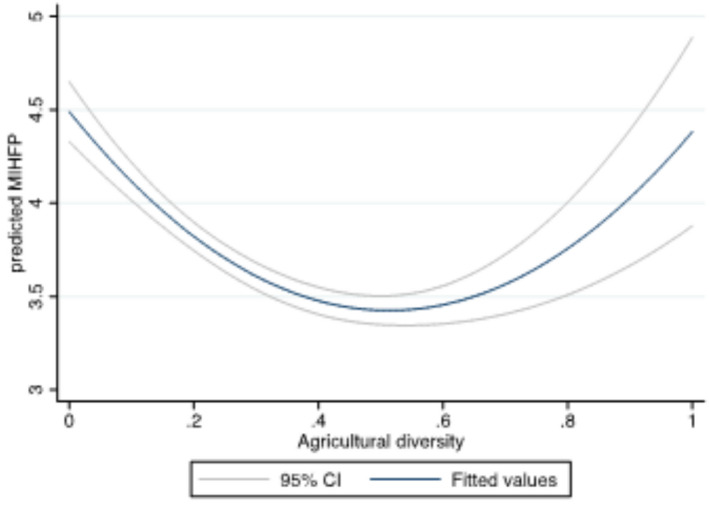
Relationship between MIHFP and agricultural diversity

As a prelude to our robust econometric analysis, we checked for bivariate relationships between our main explanatory variable (agricultural diversity index) and the dietary diversity indicators (HDDS and MIHFP) using two‐way quadratic prediction plots with confidence intervals. Without controlling for other confounding factors, HDDS has an inverse U‐shaped relationship with agricultural diversity (Figure 2), while MIHFP had a U‐shaped relationship with agricultural diversity (Figure 3). As households move from complete specialization toward diversification, the graphs suggest that households generally experience improvements in dietary diversity (Figure 2) or reduction in months with inadequate food provisioning. The key finding from these bivariate relationships is that the effect of agricultural diversity on HDDS and MIHFP is not obvious, as demonstrated by the nonmonotonic relationships. These results, however, do not provide an overall picture. The next section presents results from a multivariate framework.

### 3.2 Regression results

Regression results are provided in two tables. Table 5 presents results for the relationship between HDDS and several factors, including agricultural diversification, while Table 6 contains results for the regression of MIHFP on agricultural diversification, ceteris paribus. Each table has four columns. The first column outlines the explanatory variables, and the second reports OLS estimates, while the third and fourth columns report the outcome and selection equation results, respectively, from the Heckman two‐step model. We juxtapose the outcome equation results with the OLS results since our discussion is primarily based on these two columns.

**TABLE 5:** Regression results for HDDS using alternative specifications

Explanatory variables	OLS	Heckman Two‐step	
	HDDS	Outcome	Selection
		HDDS	Agricultural diversification dummy
(1)	(2)	(3)	(4)
Area cultivated (ha)	0.041^***^		0.082^***^
	(3.39)		(8.82)
Number of loans obtained by HH	−0.072		0.461^***^
	(−1.27)		(11.18)
Household size	0.058^***^	0.059^***^	0.020^***^
	(6.30)	(4.22)	(3.06)
Total livestock units	0.013^***^	0.062^***^	−0.097^***^
	(4.77)	(3.82)	(−21.92)
Agricultural diversification index	0.088	0.383	
	(0.46)	(0.88)	
Household head education (years)	0.136^***^	0.118^***^	−0.001
	(20.62)	(12.99)	(−0.21)
Sex of household head (1 = male)	0.016	0.113	−0.029
	(0.28)	(1.46)	(−0.75)
Household head age (years)	−0.003	−0.003	0.001
	(−1.47)	(−1.31)	(0.88)
Crop diversification extension (1 = yes)	0.396^***^	0.371^***^	0.230^***^
	(8.70)	(5.07)	(7.19)
Inorganic fertilizer used (kg)	0.000^***^	0.001^***^	−0.000^***^
	(7.50)	(9.62)	(−3.55)
Minimum tillage use (1 = yes)	−0.054	0.077	0.162^***^
	(−0.84)	(0.89)	(3.52)
Number of children under 5 years categorical variable (base category: low)			
Medium	−0.053	−0.462	0.011
	(−0.47)	(−1.41)	(0.28)
High	−0.282^**^	−0.123	0.045
	(−2.16)	(−0.34)	(0.96)
Interaction terms between number of children under 5 years categorical variable and agricultural diversification index (base category: low#agricultural diversification index)			
medium#agricultural diversification index	0.089	0.800	
	(0.32)	(1.24)	
high#agricultural diversification index	0.112	−0.228	
	(0.36)	(−0.32)	
Constant	4.462^***^	4.410^***^	−0.277^***^
	(27.38)	(12.25)	(−2.73)
Number of observations	7924	7924	
Censored observations		3856	
Uncensored observations		4068	
*R* ^2^	.214		
Adjusted *R* ^2^	.212		
Inverse mills ratio (lambda)		−0.065	
		(−0.29)	

Note

*t* statistics in parentheses ^*^ *p* < .10, ^**^ *p* < .05, ^***^ *p* < .01.

**TABLE 6:** Regression results for months of inadequate household food provisioning using alternative specifications

Explanatory variables	OLS	Heckman Two‐step	
		Outcome	Selection
	MIHFP	MIHFP	Agricultural diversification dummy
(1)	(2)	(3)	(4)
Area cultivated (ha)	−0.076^***^	−0.115^***^	0.082^***^
	(−5.08)	(−5.89)	(8.82)
Number of loans obtained by HH	0.007		0.461^***^
	(0.12)		(11.18)
Household size	0.019^*^		0.020^***^
	(1.91)		(3.06)
Total livestock units	−0.013^***^		−0.097^***^
	(−5.92)		(−21.92)
Agricultural diversification index	−0.297	−0.062	
	(−1.38)	(−0.14)	
Household head education (years)	−0.069^***^	−0.058^***^	−0.001
	(−10.78)	(−6.20)	(−0.21)
Sex of household head (1 = male)	−0.302^***^	−0.265^***^	−0.029
	(−4.74)	(−3.32)	(−0.75)
Household head age (years)	0.005^***^	0.007^***^	0.001
	(2.84)	(2.92)	(0.88)
Crop diversification extension (1 = yes)	−0.239^***^	−0.289^***^	0.230^***^
	(−4.84)	(−4.20)	(7.19)
Inorganic fertilizer used (kg)	−0.000^***^	−0.000^***^	−0.000^***^
	(−3.90)	(−2.80)	(−3.55)
Minimum tillage use (1 = yes)	−0.167^***^	−0.328^***^	0.162^***^
	(−2.67)	(−3.77)	(3.52)
Number of children under 5 years categorical variable (base category: low)			
Medium	0.194	0.425	0.011
	(1.52)	(1.27)	(0.28)
High	0.131	−0.206	0.045
	(0.90)	(−0.55)	(0.96)
Interaction terms between number of children under 5 years categorical variable and agricultural diversification index (base category: low# agricultural diversification index)			
medium# agricultural diversification index	−0.216	−0.613	
	(−0.70)	(−0.92)	
high# agricultural diversification index	0.183	0.867	
	(0.53)	(1.18)	
Constant	1.938^***^	2.187^***^	−0.277^***^
	(11.51)	(6.49)	(−2.73)
Number of observations	7924	7924	
Censored observations		3856	
Uncensored observations		4068	
*R* ^2^	.113		
Adjusted *R* ^2^	.110		
Inverse mills ratio (lambda)		−0.313^**^	
		(−2.01)	

Note

*t* statistics in parentheses ^*^ *p* < .10, ^**^ *p* < .05, ^***^ *p* < .01.

The main interest of this paper is to estimate the effect of agricultural diversity on HDDS and MIHFP across three categories of households based on the number of children that are 5 years and younger. Therefore, we are interested in interpreting the parameter estimates on both the SI for agricultural diversity variable and the interaction terms between agricultural diversity and the dummy variables encapsulating the number of underfive children in each household. However, we highlight other variables that statistically significantly affect the two indicators of interest in this study. Although our regression analysis includes fixed effects controlling for the 10 provinces in Zambia, we do not report the point estimates for these variables.

First, we highlight the key findings of the relationship between HDDS and agricultural diversity conditional on other factors (Table 5). A positive and statistically significant parameter estimate on the agricultural diversification index variable would imply that households that diversify agricultural production are more likely to improve household dietary diversity. Based on our OLS and outcome equation results (Heckman model), the relationship mentioned above is upheld as demonstrated by the positive coefficients on the diversification index for the two specifications. Interestingly, the magnitude of the coefficient agricultural diversity index (0.383) in the Heckman outcome equation is more than four times that in the OLS regression (0.088). However, the results for both specifications are not statistically significant, implying that while agricultural diversification is positively associated with HDDS (as conjectured in the literature), the association is not enough to uphold a strong linkage between the two variables in Zambia. In the context of the extant literature, several studies have found a positive and statistically significant link between agricultural diversification and dietary diversity (e.g., Kumar et al., 2015; Hossain et al., 2016; Mazunda et al., 2015).

Apart from looking at the relationship between agricultural diversity and household dietary diversity, we were also interested in unpacking how this relationship differs across the distribution of households based on the number of underfive children. To implement this, we include interaction terms between the agricultural diversification index and a categorical variable that divides the number of children under 5 years in the sample into terciles (three groups). Specifically, the interaction terms are as follows:

- Dummy variable for a low number of underfive children interacted with the agricultural diversification index.
- Dummy variable for medium number of underfive children interacted with the agricultural diversification index.
- Dummy variable for high number of underfive children interacted with the agricultural diversification index.

Using the first dummy variable as our base category, the interaction terms test the following hypothesis: The relationship between agricultural diversification and HDDS differs across the distribution of households based on the number of underfive children. Across the two model specifications, we do not have strong evidence to suggest that households with more children under the age of five and diversify agricultural production are more likely to have better outcomes of household dietary diversity.

Other factors that are significant and positively associated with HDDS are land cultivated, household size, livestock units, household head education, households receiving extension information, and use of productivity‐enhancing inputs like fertilizers. Further, these results are robust across the two specifications. For the most part, the pathway to improved dietary diversity is consistent with the findings of other studies. For instance, Hossain et al. (2016) found that household income, land ownership, the level of education of the head and the spouse, the sex of the household head, and the level of infrastructure development as measured by access to electrification play a significant role in improving dietary diversity in Bangladesh.

Regression analysis of household‐level data from Indonesia, Kenya, Ethiopia, and Malawi shows that agricultural diversity was not always positively associated with dietary diversity. In fact, the association was sometimes negative when agricultural diversity was already high, which was attributed to income losses from a lack of specialization (Sibhatu et al., 2015). The study also highlighted that market access and transactions had a more significant impact on dietary diversity than increased agricultural diversity.

For the second set of regression results (Table 6), a negative and statistically significant parameter estimate on the agricultural diversification index variable would be interpreted as follows: A marginal increase in agricultural diversification leads to a decrease in the number of months with inadequate household food provisioning. For both specifications, the parameter estimates suggest that agricultural diversification is negatively associated with MIHFP. Despite this a priori outcome, the results are not statistically significant, implying that the association is just random and not a confirmation of a strong link between the two variables in our data. Furthermore, the statistically insignificant coefficients on the interaction terms suggest that there are no significant differences in the number of months with inadequate household provisioning due to changes in agricultural diversification as one compares households across the distribution of the number of children under 5 years. Finally, besides the dummy variable minimum tillage use (1 = yes), the list of factors that have a more than random contribution to improved food security (decrease in MIHFP) is similar to those affecting HDDS.

## 4 CONCLUSIONS AND IMPLICATIONS FOR POLICY

In contrast to predicted findings, agricultural diversification in Zambia was not strongly associated with improving household dietary diversity or months during which households have inadequate food provisioning. Also, the number of children under 5 years in each household did not appear to impact dietary diversity. It may be possible that nonsociodemographic factors that were not measured in the dataset (e.g., intrahousehold food distribution) could have affected household dietary diversity. Theoretically, agricultural diversification should lead to improved individual nutrition outcomes. Unfortunately, it was impossible to test this hypothesis as the RALS survey did not collect information on individual‐level dietary intake. The study could have also benefited from data on the frequency of household consumption and nutritional status.

Factors including land cultivated, household size, livestock units, household head education, households receiving extension information, and use of fertilizers were all positively associated with household dietary diversity. This indicates that these factors may contribute to an increase in income and hence increased household finances and the ability to purchase a more diverse range of foods. Therefore, our study suggests that access to income may have a more significant role in achieving household dietary diversity than agricultural diversification.

It also highlights the need for policy initiatives that improve access to income and inputs among women farmers. In addition, with the relatively long distances between farmers and urban centers, improving access to urban markets by investing in rural transport systems should also be investigated from a policy perspective.

Policy interventions that focus on increasing farm productivity and hence access to income and behavior change communication to provide information on the importance of consuming a diverse diet could improve household dietary diversity. Further investigations are warranted to confirm the feasibility of these recommendations.

## CONFLICT OF INTEREST

The authors declare no conflict of interest with this publication.

## AUTHOR CONTRIBUTIONS

**Chewe Nkonde:** Conceptualization (equal); Data curation (equal); Formal analysis (lead); Funding acquisition (supporting); Investigation (equal); Methodology (equal); Project administration (supporting); Resources (supporting); Software (equal); Supervision (equal); Validation (equal); Visualization (equal); Writing‐original draft (supporting); Writing‐review & editing (equal). **Keiron Audain:** Conceptualization (equal); Data curation (equal); Formal analysis (supporting); Funding acquisition (equal); Investigation (equal); Methodology (supporting); Project administration (lead); Resources (equal); Software (supporting); Supervision (equal); Validation (supporting); Visualization (equal); Writing‐original draft (lead); Writing‐review & editing (lead). **Pamela Marinda:** Conceptualization (equal); Data curation (equal); Formal analysis (supporting); Funding acquisition (lead); Investigation (equal); Methodology (supporting); Project administration (supporting); Resources (lead); Software (equal); Supervision (equal); Validation (equal); Visualization (equal); Writing‐original draft (supporting); Writing‐review & editing (supporting). **Rebecca N. Kiwanuka‐Lubinda:** Conceptualization (equal); Data curation (supporting); Formal analysis (equal); Funding acquisition (equal); Investigation (supporting); Methodology (supporting); Project administration (supporting); Resources (supporting); Software (equal); Supervision (equal); Validation (equal); Visualization (equal); Writing‐original draft (supporting); Writing‐review & editing (supporting).

## ETHICAL STATEMENT

This study analyzed secondary data prepared by The Indaba Agricultural Policy Research Institute (IAPRI). Findings included in their report were based on the nationally representative Rural Agricultural Livelihood Survey (RALS) conducted in Zambia between June and July 2015, in collaboration with the Central Statistical Office (now the Zambia Statistics Agency) and the Ministry of Agriculture. The Zambia Statistics Agency is mandated by law to conduct nationwide surveys and hence is exempted from applying for ethical approval.

## STUDIES INVOLVING HUMAN OR ANIMAL SUBJECTS

This article does not contain any studies with human or animal subjects performed by any of the authors.

## Data Availability

The data that support the findings of this study are available on request from Indaba Agricultural Policy Research Institute at https://www.iapri.org.zm/rals‐2015‐data/
